# Deciphering the molecular mechanisms of stem cell dynamics in hair follicle regeneration

**DOI:** 10.1038/s12276-023-01151-5

**Published:** 2024-01-05

**Authors:** Jung Hyun Lee, Sekyu Choi

**Affiliations:** 1 Department of Dermatology, School of Medicine, University of Washington, Seattle, WA 98109 USA; 2 Institute for Stem Cell and Regenerative Medicine, University of Washington, Seattle, WA 98109 USA; 3 Department of Life Sciences, Pohang University of Science and Technology (POSTECH), Pohang, 37673 Republic of Korea; 4 Medical Science and Engineering, School of Convergence Science and Technology, Pohang University of Science and Technology (POSTECH), Pohang, 37673 Republic of Korea; 5 School of Interdisciplinary Bioscience and Bioengineering, Pohang University of Science and Technology (POSTECH), Pohang, 37673 Republic of Korea; 6 Institute for Convergence Research and Education in Advanced Technology (I_CREATE), Yonsei University, Incheon, 21983 Republic of Korea

**Keywords:** Skin stem cells, Stem-cell niche, Skin stem cells

## Abstract

Hair follicles, which are connected to sebaceous glands in the skin, undergo cyclic periods of regeneration, degeneration, and rest throughout adult life in mammals. The crucial function of hair follicle stem cells is to maintain these hair growth cycles. Another vital aspect is the activity of melanocyte stem cells, which differentiate into melanin-producing melanocytes, contributing to skin and hair pigmentation. Sebaceous gland stem cells also have a pivotal role in maintaining the skin barrier by regenerating mature sebocytes. These stem cells are maintained in a specialized microenvironment or niche and are regulated by internal and external signals, determining their dynamic behaviors in homeostasis and hair follicle regeneration. The activity of these stem cells is tightly controlled by various factors secreted by the niche components around the hair follicles, as well as immune-mediated damage signals, aging, metabolic status, and stress. In this study, we review these diverse stem cell regulatory and related molecular mechanisms of hair regeneration and disease conditions. Molecular insights would provide new perspectives on the disease mechanisms as well as hair and skin disorder treatment.

## Stem Cells: The Secret Behind Continuous Hair Regeneration”

Hair follicles are complex structures in the skin that play key roles in functions like hair growth and wound healing. However, our knowledge of how hair follicle stem cells (HFSCs) can divide and develop into different cell types, and their potential in hair regeneration is still limited. In this study, researchers reviewed the internal and external processes involved in the regulation of HFSCs, melanocyte stem cells (MeSCs), and sebaceous gland (SG) stem cells during the hair growth cycle. The study also examined the effects of aging, metabolic changes, and stress on hair follicle regeneration. The researchers concluded that understanding the stem cell regulation-related signaling pathways in the hair follicles could be crucial for developing strategies for hair regeneration and the treatment of various hair and skin disorders.

This summary was initially drafted using artificial intelligence, then revised and fact-checked by the author.

## Introduction

Hair follicles (HFs) are complex structures within the skin with crucial roles in various functions, such as thermoregulation and sensory input detection^1^. They comprise actively dividing progenitor cells in the hair matrix, which proliferate and develop into differentiated progeny, and hair follicle stem cells (HFSCs), which undergo dynamic molecular changes necessary for hair growth promotion and wound healing promotion in the skin^2–4^. HFSCs are regulated by internal and external signals, which determine their behaviors^3,5^. Transcription factors and key signaling pathways, such as the BMP, FOXC1, NFATC1, Shh, and Wnt pathways, are critical for regulating HFSC behaviors^2,6–10^. The dermal papilla (DP), comprising mesenchymal cells, regulates HF growth, and epithelial-mesenchymal interactions are pivotal for maintaining morphogenesis and the hair growth cycle^11^. While progress has been made in developing approaches to target the regulatory mechanisms of the DP and various HFSC niches in the promotion of HF growth^12,13^, further research is needed for an in-depth understanding of their potential in complete HF regeneration.

The immune privilege (IP) of anagen HFs is a unique characteristic that protects follicles from immune attacks^14^. Several inflammatory hair loss disorders have been linked to HF-IP dysfunction and loss^15,16^. Hormones, such as androgens, estrogens, and prolactin, are also central to the regulation of the hair growth cycle^17–19^. Increased androgen or prolactin levels, as well as hormonal imbalances, are closely linked to baldness, also known as alopecia^20,21^. Exploring the immune response regulatory mechanisms within the HF microenvironment and the hormonal effects in regulating hair growth is vital to addressing these conditions^22–24^.

Melanocytes in the skin play a significant role in determining skin and hair color as well as safeguarding against ultraviolet (UV)-induced DNA damage. Understanding the gene expression and signaling pathway regulation in melanocyte stem cells (MeSCs) in niches is thus essential for developing effective strategies to enhance skin health and prevent skin pigment disorders^25^. Furthermore, environmental factors, such as UV radiation, can also affect melanocyte behavior and alter gene expression, leading to pigmentation-related changes and an increased risk of skin cancer^26^. Sebaceous glands (SGs) are HF-associated sebum lipid-producing essential epidermal structures that contribute to barrier function and microbiome composition in the skin^27^. Excess and reduced sebum production result in problems such as oily skin and eczematous diseases, respectively^28^. Moreover, androgenic alopecia (AGA), a common hair loss condition, is characterized by SG hypertrophy^29^. Therefore, further research is needed to fully understand the regulatory mechanism of MeSC and SG stem cell dynamics in the hair growth cycle and identify novel targets for therapeutic interventions.

This review delves into the complex molecular and cellular processes involved in niche-related dynamic skin stem cell behaviors in HFs. Specifically, we summarized HFSC, MeSC, and SG stem cell functions and activities during hair growth cycles. We aimed to provide a comprehensive analysis of the immune-mediated damage process in HFs as well as the effects of aging, metabolic changes, and stress on HF regeneration. A detailed overview of these aspects could encourage future research and provide new insights into hair regeneration.

## Stem cell dynamics in the hair follicle

The HF is a unique miniature organ in mammalian skin that undergoes continuous regeneration cycles comprising the anagen, catagen, and telogen phases^1,30^. The anagen phase in the human scalp frequently lasts for 2–6 years, whereas in mouse dorsal skin, it normally lasts for approximately 2 weeks^31^. Due to its characteristics, the mouse HF is a useful model for studying stem cell quiescence and activation^32,33^. During the telogen phase, a quiescent HFSC population resides in the outer layer of the bulge region, an anatomical niche, while primed HFSCs are located in the hair germ^2,3^. HFSCs remain quiescent for most of the hair growth cycle, except for the early anagen phase when they become active. During the telogen-to-anagen transition, primed HFSCs in the hair germ are the first cells to become activated before bulge HFSC activation^3^. The anagen phase begins when the quiescent signals from the inner bulge layer and other HFSC niches are overwhelmed by a threshold level of the combined BMP inhibitory and Wnt activating signals^3,6,34–36^. These primed HFSCs generate transit-amplifying cells (TACs), rapidly proliferating progenitor cells located in the hair matrix^2^. The DP regulates the lineage choices of TACs throughout the progression of the anagen phase^37^. Following terminal differentiation, TACs give rise to downstream HF lineages, including the hair shaft, companion layer, and inner root sheath^38^. On the other hand, bulge HFSCs mainly give rise to the downward-growing outer root sheath^34^. During the catagen phase, the lower portion of the HF regresses with a gradually shortening epithelial strand before entering the upcoming telogen phase^39^.

MeSCs are undifferentiated cells that originate from the neural crest and reside in the bulge and hair germ area of the HF^40^. During the telogen phase, MeSCs remain quiescent until anagen onset. In the early anagen phase, activated MeSCs generate proliferating progenitors that migrate to the hair bulb and differentiate into melanin-producing melanocytes, which are responsible for the color of the newly formed HFs^41^. During the catagen phase, differentiated melanocytes undergo apoptosis within the hair matrix^42^. However, when exposed to UV radiation, MeSCs can migrate toward the epidermis and differentiate into melanocytes, contributing to epidermal pigmentation as a protective function^43^.

The SG maintains its connection to the HF structure, located in the upper permanent HF region, specifically in the junctional zone where Lrig1^+^ SG stem cells reside^44^. SCD1^+^ proliferative SG progenitor cells are continuously produced within the SG outer layer. These cells subsequently differentiate into mature sebocytes filled with lipids in the inner layer^45^. Mature sebocytes undergo a unique form of programmed cell death during the holocrine secretion of lipid-containing sebum, in which their cellular organelles are degraded^46^. Sebum is released through the sebaceous secretory duct of the SG into the HF canal, ultimately reaching the skin surface^28,47^. However, disrupted SG homeostasis, such as that seen in sebaceous hyperplasia, could affect HF–SG communication^48^.

## Stem cell regulatory mechanisms in the hair follicle niche

**Fig. 1:**
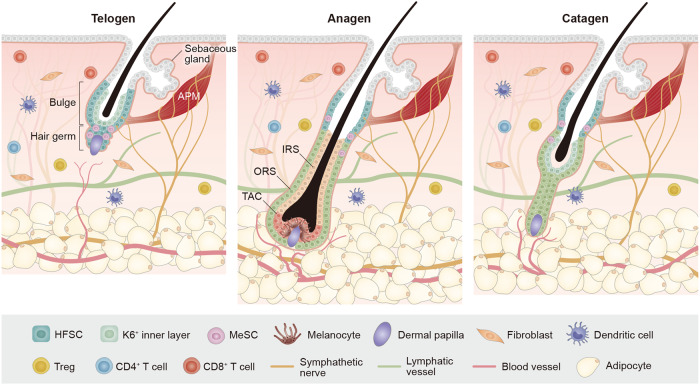
Skin stem cells and niches orchestrate the hair growth cycle, encompassing the telogen, anagen, and catagen phases. Hair follicle stem cells can generate various cell types within the HF. The immune system, including T cells, macrophages, dendritic cells, and Treg cells, undergoes fluctuations during the hair growth cycle and plays an important role in HF regeneration. Blood vessels play a crucial role in HF regeneration by providing oxygen, nutrients, and growth factors essential for hair growth and maintenance. The surrounding vasculature enables the delivery of these vital components. The interactions among lymphatic vessels, sympathetic nerves, sensory nerves, and HFs are complex and interconnected, collectively contributing to HF function regulation, including nutrient supply, immune surveillance, and responses to environmental cues.

HFSCs are sustained by niches, specialized microenvironments that regulate their functions^13,49,50^. The niches are composed of neighboring cells that produce various factors, impacting HFSCs both directly and indirectly^36,51,52^. One of the most well-known HFSC niches is the DP, a mesenchymal cell cluster that modulates HFSC activation, proliferation, and differentiation through multiple signals^32^. Other niches include those of adipocyte precursor cells, mature dermal adipocytes, dermal macrophages, regulatory T cells, blood vessels, lymphatic vessels, and sympathetic nerves^53–55^ (Fig. 1). These niches secrete signals that either activate HFSCs or maintain their quiescence during the hair growth cycle^12^.

Several signaling pathways, such as the BMP, FGF, Wnt, and JAK/STAT pathways, mediate niche factor effects on HFSC activity. BMP signaling maintains HFSC quiescence during early telogen, and premature HFSC activation occurs upon BMP receptor 1A ablation^56,57^. In addition, upregulation of DP-derived BMP antagonists, such as Noggin, Bambi, or Sostdc1, promotes the early-to-late telogen transition in adult HFs^3,6^. Additionally, FGF7 and FGF10 upregulation within the DP in late telogen stimulates HFSC activation in the hair germ located above the DP^3^. FGF18 is highly expressed throughout the telogen phase and secreted from the inner bulge layer, regulating the HFSC quiescent state^34,58^. FGF9 from dermal γδ T cells is important in the wound-induced HF neogenesis process^59^. Pharmacological inhibition of the JAK-STAT pathway enhances hair growth by disrupting HFSC quiescence^60^. Dermal TREM2^+^ macrophage-produced oncostatin M regulates HFSC quiescence by acting upstream of JAK-STAT5 signaling^61^. Wnt/β-catenin signaling promotes HFSC activation and HF growth^10,62^. However, DP cell stimulation with dihydrotestosterone prevents HFSC differentiation by increasing the expression of the Wnt inhibitor Dkk-1^63^.

The identity of HFSCs and the capacity for HF regeneration are governed by cell-intrinsic molecular factors, such as transcription factors and epigenetic modifiers that control gene expression (Table 1). These cell-intrinsic molecules may function as downstream or upstream signaling pathway targets to coordinate the effects of niche factors on HFSC activity as well as HF growth^5,36^. Several transcription factors have been found to influence HFSC quiescence, self-renewal, directional migration, or lineage fate specification during the hair growth cycle^7–9,64–76^. Additionally, chromatin remodeling factors can affect HFSC behaviors. The histone modification activity of polycomb repressive complex 1 (PRC1) is necessary to maintain LGR5^+^ HFSCs and HF regeneration^77^. Loss of DNA methyltransferase 1 (DNMT 1) results in delayed HFSC activation after plucking and reduced proliferation of HF progenitor cells^78^. Further study is needed to elucidate the intricate molecular mechanisms by which transcription factors and epigenetic modulators interact with HFSC niches, thus regulating the expression of genes specific to the hair growth cycle.

**Table 1:** Functions of cell-intrinsic molecules in controlling HFSC behaviors.

Molecule	Description	Refs.
NFATC1	Maintains HFSC quiescence by repressing CDK4 activity Regulates JAK/STAT pathway in HFSCs during pregnancy	^9,133^
TCF3/4	Maintains HFSC quiescence by repressing Wnt/β-catenin signaling	^64^
FOXC1	Maintains HFSC quiescence through activating NFATC1 and BMP signaling	^7,8^
LHX2	Maintains HFSC quiescence and organize the bulge niche	^65^
FOXP1	Regulates HFSC quiescence through the modulation of FGF18	^66^
RUNX1	Regulates HFSC activation and promotes HFSC proliferation by repressing p21 and p15	^67,134^
IRX5	Promotes HFSC activation by repressing FGF18 expression	^68^
OVOL2	Facilitates directional migration of bulge HFSCs by repressing the expression of the EMT activator ZEB1	^69^
FOXI3	Regulates HFSC specification and is essential for the process of HF downgrowth	^70^
SOX9	Regulates HFSC maintenance Binds HFSC-specific super-enhancers to modulate lineage status	^71,135^
LGR4	Promotes the hair growth cycle by activating HFSCs	^72^
LGR5	Promotes stem cell self-renewal Induces Shh signaling to drive proliferative status during the anagen phase	^73,136^
GATA6	Prevents DNA damage and apoptosis in HF progenitor cells Essential for normal proliferation of HF progenitor cells	^75^
LEF1	Regulates HFSC lineage fate specification	^64,74,137^
NFI	Maintains the chromatin landscapes of bulge HFSCs	^76^
HES1	Modulates Shh signaling induction in HFSC activation	^138^
SIRT7	Deacetylates NFATC1, causing the activation of HFSCs	^139^
PRC1	Monoubiquitinates lysine 119 of histone H2A (H2AK119Ub) and controls the transcriptional landscape in HFSCs	^77^
DNMT1	Maintains activation of HFSCs and proliferation of HF progenitor cells	^78^

MeSCs reside in the same niches as HFSCs and undergo periodic rest, activation, and degeneration phases, coinciding with HFSCs, for hair pigmentation^79^. The quiescent MeSC state depends on the role of niche-derived TGF-β signaling^80^. Adjacent HFSCs in the hair germ produce Wnt ligands, including Wnt10b, with the potential to activate MeSCs. Moreover, MeSC activation during early anagen relies on the significance of Wnt/β-catenin signaling^79^. Interestingly, neighboring HFSCs secrete endothelin-1 (Edn1) during anagen onset, and HFSCs exhibit increased Edn2 expression upon the removal of the NFIB transcription factor^79,81^. The Edn/EdnrB pathway affects MeSC proliferation and maintenance^82^. The ligand for c-KIT, stem cell factor (SCF) secreted from the hair matrix and DP is critical for melanocyte maintenance^83,84^. When exposed to UV irradiation, MeSCs from HFs migrate to the epidermal layer through melanocortin 1 receptor signaling and differentiate into functional melanocytes to protect damaged skin^43^.

SG regulation is a complex process encompassing both local mediators and potential long-range hormonal signals^85^. Multiple signaling pathways, including the Notch, Wnt, FGF, retinoid and aryl hydrocarbon receptor pathways, mediate the effects on SG stem cell activity. However, our understanding of the SG regulatory niche remains limited. Notch signaling directly promotes SG stem cell differentiation and indirectly inhibits these glands outside the stem cell population^85^. FGF signaling influences SG homeostasis, as proven by SG atrophy in the tail skin of mutant mice lacking Fgfr2b^86^. Retinoids can treat acne by shrinking SGs and reducing sebum production^87^. The immune system-mediated regulation of sebum secretion is noteworthy. Thymic stromal lymphopoietin (TSLP), an epithelial cell-produced cytokine, stimulates the migration of T cells to SGs, which is necessary for increased sebum secretion^88^. The absence of skin-resident innate lymphoid cells (ILCs) leads to SG hyperplasia due to the lack of ILC-derived TNF and lymphotoxin production, mediating the antiproliferative effect on sebocytes by Notch signaling inhibition^89^.

In conclusion, dysfunctional HF niches negatively impact the hair growth cycle, pigmentation, or sebum production by influencing stem cell functions in HFs. Therefore, understanding the stem cell regulation-related signaling pathways and niche factors in HFs is important to developing strategies for HF regeneration and the treatment of various hair and skin disorders.

## Immune privilege in hair follicle growth and regeneration

**Fig. 2:**
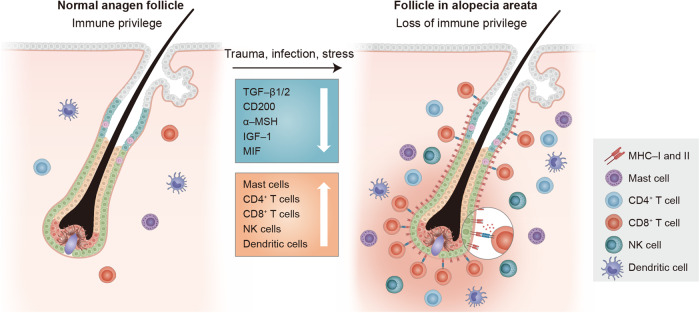
Loss of hair follicle-immune privilege in alopecia areata. Immune cell populations can be found around HFs in a typical hair growth cycle. However, when alopecia areata develops, the anagen phase is significantly reduced, resulting in disordered HF growth. This is accompanied by a characteristic inflammatory cell infiltration in the HF-surrounding area, involving CD4^+^ and CD8^+^ T cells, mast cells, NK cells, and dendritic cells. Typically, CD8^+^ T cells are the first to infiltrate the intrafollicle, followed by dendritic cells and macrophages. CD4^+^ T cells are rarely found within HFs until late in the disease process. The collapse of HF-IP plays a central role in AA pathogenesis, involving factors such as MHC molecules, cytokines, costimulatory molecules, Tregs, and perifollicular cells that contribute to IP maintenance, disruption, and restoration.

HF-IP is critical for maintaining the hair growth cycle and preventing immune-mediated HF damage^90,91^. This protective mechanism remains active during the anagen phase and restricts immune surveillance^14^. Alopecia areata (AA) is a form of nonscarring hair loss in humans characterized by HF-IP breakdown and lymphocyte infiltration around the anagen bulb^16^. In AA, HFs undergo dystrophy and transition into the catagen and telogen phases, resulting in a reduced proportion of anagen HFs^16^. MHC class I and II molecules (MHC-I and MHC-II, respectively), which are involved in immune cell-related foreign antigen recognition, are reduced in the bulge and hair bulb during HF-IP^92–96^. HF-IP effectively suppresses the immune cell response by establishing an immunosuppressive signaling environment in anagen HFs, facilitated by significant CD200 expression in the bulge^97,98^. In addition, other mediators produced by HFs, including neuropeptides (*α*-MSH), growth factor family members (TGF-*β*1/2, IGF-1), and macrophage migration inhibitory factor, contribute to CD8^+^ T-cell and natural killer cell suppression^99–103^. These mediators work together to maintain a stable hair growth cycle by preventing the immune system from attacking HFs^14,97^ (Fig. 2).

Recent research using “just EGFP death-inducing” (JEDI) mouse models revealed an interesting relationship between stem cell quiescence and immune system evasion ability. JEDI T cells effectively eliminated GFP^+^ intestinal, mammary gland, and ovarian stem cells. By reducing MHC-I and NLRC5 expression, Lgr5^+^ quiescent HFSCs in the bulge region exhibited complete resistance to T-cell-mediated attack^104^. Primary cicatricial alopecia (PCA), including frontal fibrosing alopecia and lichen planopilaris (LPP), is a scarring form of hair loss in humans, distinguished by the presence of a perifollicular lymphocytic infiltrate and inflamed HF replacement with fibrous tissue^105^. Lesional LPP skin can be characterized by the loss of IP in bulge HFSCs, increased MHC-I and MHC-II expression and reduced CD200 and TGFβ2 expression^23,96^. According to recent research, nuclear factor IB (NFIB) and IX (NFIX) are essential for maintaining the identity of HFSCs, and their removal causes immune infiltration phenotypes in Nfib/Nfix double conditional knockout mice, which resemble PCA^76^. Consequently, understanding HF-IP-related mechanisms is essential for developing effective treatments against hair loss disorders.

## The impact of aging, metabolism, and stress on hair growth and regeneration

**Fig. 3:**
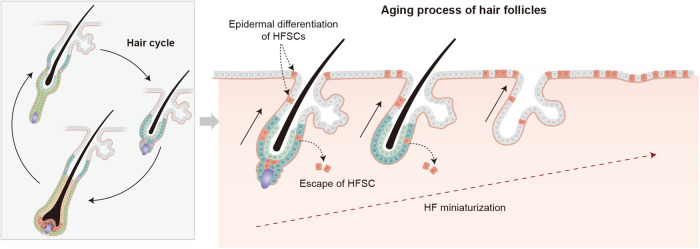
Mechanisms for hair follicle aging. During aging, hair follicle stem cells transition into a quiescent phase, and the niches undergo age-related changes. In aging skin, the regenerative ability of HFSCs becomes impaired. The niche is vital in various aging-related processes, including epidermal differentiation and HF miniaturization. HF aging is characterized by the miniaturization and absence of the dermal papilla. Prior to miniaturization, stem cells undergo dysregulation, including the loss of HFSC markers and maintenance markers. HFSCs undergo epidermal terminal differentiation and escape from the bulge during aging.

HF growth and regeneration are complex processes influenced by various factors, including aging and metabolism, which could impact HFSC quiescence and activation as well as hair shaft miniaturization^106,107^. In androgenic alopecia, HFSC loss-associated progressive HF miniaturization can be observed^107–109^. Regenerative HF domains reportedly become asynchronous with aging^110^, and HFSCs become increasingly quiescent^7,111,112^, leaving their HF niche^108^ or differentiating into epithelial keratinocytes during aging^107^ (Fig. 3). Importantly, not only aged HFSCs but also their niches may contribute to these changes^113,114^. Therefore, understanding the complex interactions between HFSCs and their niches during aging and metabolic disorders is essential for developing effective hair regeneration promotion and hair loss treatment strategies.

Recent research investigating the role of the skin niche during aging has yielded valuable insights into the promotion of HF regeneration. The substantial changes in the HFSC niches leading to altered HFSC behavior and declining skin regeneration ability have been elucidated^113^. The study revealed that aged HFSCs could regenerate HFs in the presence of young dermis^113,114^, while young HFSCs could only regenerate HFs when supported by young dermis^113^. These results indicate that niche aging is key to determining stem cell behavior. Moreover, age-related changes were observed in various nonepithelial niche cell types, including immune cells, dermal fibroblasts, and sensory neurons^113^. In addition, increased cellular identity noise and a gain of proadipogenic characteristics in dermal fibroblasts could be potential underlying mechanisms of aging^115^. With aging, the hemidesmosomal collagen COL17A1, a basement membrane anchor for HFSCs, is depleted, thereby contributing to HF miniaturization and thinning^107^. Furthermore, extracellular matrix (ECM) component expression-related changes could increase basement membrane thickness and stiffness, resulting in the suppression of important HFSC activation promoters in aged HFs^114^.

In addition to skin niche alternations, systemic and cellular metabolic changes could also impact hair growth and regeneration. High-fat diet-induced obesity reportedly accelerates HF thinning and loss by suppressing Shh signaling and inducing HFSC differentiation into epithelial keratinocytes^116^. The importance of several metabolic pathways in HFSC activation has also been revealed (e.g., lactate production is potentially essential for HFSC activation)^117^. In addition, glutamate signaling is significant in HFSC activation, as the glutamate transporter Slc1a3 is critical for anagen entry^118^. Ceramide synthase 4 inactivation reportedly caused HFSC activation, suggesting that epidermal ceramide composition is significant in regulating HFSC behaviors^119^. Furthermore, inhibiting glutamine metabolism is necessary for progenitor cells to return to the quiescent HFSC state^120^.

Hair graying is among the most obvious aging phenomena in mammals. The MeSC number reportedly decreases, and MeSCs undergo premature differentiation in aged HFs^121,122^. In addition, ionizing radiation (IR)-induced genotoxic stress induces premature hair graying in mice. The ATM-mediated DNA damage response plays a critical role in determining MeSC fate, preventing premature differentiation^122^. Recently, organ-cultured human HFs have shown that exposure to IR results in pigmentation loss, establishing accumulated DNA damage as an important contributing factor in human hair graying^123^. Under resiniferatoxin-induced acute stress in mice, the sympathetic nervous system becomes hyperactivated, resulting in significant norepinephrine release, inducing the entry of MeSCs to a rapid proliferation state. These MeSCs migrate, differentiate, and eventually deplete from the bulge. The β2 adrenergic receptor on MeSCs is a critical molecule in stress-induced hair-graying mice^124^. However, whether MeSC loss during the aging process is influenced by DNA damage or the sympathetic nervous system remains unclear and needs further elucidation.

Chronic stress is reportedly a contributing factor to hair loss. Stress-induced changes in cellular signaling pathways and corticosterone inhibitory effects on hair regrowth have also been observed^112^. Chronic stress reportedly inhibits HFSC activation by reducing *Gas6* expression. Restoring *Gas6* levels reversed the effects of chronic stress and promoted hair growth in mice. Suppressing corticosterone (the mouse equivalent of cortisol) signaling could activate HFSCs and alleviate the negative impact of stress on hair growth.

## Perspectives

In this study, we reviewed our current understanding of various skin stem cell-related regulatory aspects within their niches to achieve hair regeneration. Since these stem cells or niches are affected by factors such as immune dysfunction, aging, metabolic changes, and stress, finding the right target for each condition is important for overcoming hair loss, which causes psychological distress in many people.

Several hair loss treatments have already been developed. Among them, noninvasive treatments such as low-level laser therapy and microneedling therapy reportedly stimulate partial hair growth^125,126^. Low-level laser therapy works by stimulating HF activity and promoting hair growth through photobiomodulation, a process that involves light energy absorption by the cells^125,127^. Microneedling therapy is another novel treatment involving the creation of small puncture wounds on the scalp with a tiny needle-bearing device, thereby stimulating hair growth by increasing blood flow to the scalp and promoting growth factor release^126^. However, the underlying stem cell- and niche-specific regulatory mechanisms of these methods remain unknown.

In addition to noninvasive treatments, several hair loss treatment drugs are also available. Finasteride is an FDA-approved drug that inhibits 5-alpha reductase, an enzyme responsible for testosterone-to-dihydrotestosterone conversion, and is effective in reducing hair loss^128^. By reducing dihydrotestosterone production, this medication could help slow or reverse hair loss in individuals with male-pattern baldness^129^. JAK inhibitors have also gained attention as a potential treatment for AA, an autoimmune disorder^130–132^. These medications work by blocking the activity of JAK enzymes, which are involved in immune system function and inflammation.

Taken together, the existence of these treatments provides hope for individuals experiencing hair loss. However, the efficacy of these treatments still differs depending on the underlying cause of hair loss and personal factors. However, further understanding of the skin stem cell and niche regulation processes that affect hair growth and reverse alopecia is needed for developing improved treatment approaches.
